# The optimization of superficial planning target volumes (PTVs) with helical tomotherapy

**DOI:** 10.1120/jacmp.v15i6.4560

**Published:** 2014-09-08

**Authors:** Mark J. Ashburner, Samuel Tudor

**Affiliations:** ^1^ Medical Physics Addenbrooke's Cambridge UK

**Keywords:** Inverse treatment planning, fluence, build‐up region, IMRT, TomoTherapy

## Abstract

When the planning target volume (PTV), extends close to or beyond the skin, inverse‐planned intensity‐modulated radiotherapy can boost the fluence from tangential angles to superficial parts of the PTV due to the buildup effect. This can result in significant regions of high dose upon a small movement in the patient's position. We analyzed different methods of reducing this effect in the TomoTherapy planning system, including using pretend bolus, clipping the PTV back from the skin surface, and combinations of both. No alternative technique was seen to be unconditionally superior to a 5 mm planning bolus. A clip distance of 3 mm gave acceptable results, but consideration should be given to the reduction in minimum dose or increase in maximum dose seen in clip distances just 1 mm less or more, respectively, when treating head and neck cases in TomoTherapy.

PACS numbers: 87.55.de, 87.55.dh

## I. INTRODUCTION

In situations where the clinical target volume (CTV) extends to near the skin surface, the planning target volume (PTV), can extend up to and beyond the skin, a situation commonly seen for the primary or nodal target volumes in the treatment of head and neck cancers. When inverse‐planned intensity‐modulated radiotherapy (IMRT) is the chosen modality, fluence boosting effects occur as inverse‐planning systems try to compensate for a lack of buildup material at the patient's skin surface. In situations where the PTV extends up to or near the surface of the patient, the treatment planning system compensates by boosting beamlets tangential to the superficial region. When the patient is displaced from their planned position, notable regions of high dose are presented upon a small movement in the patient's position, even when the original plan shows good homogeneity.^1^, ^2^, ^3^ Such an effect is distinct from other potential sources of increased superficial dose, such as the bolus effect of immobilization masks.^(4)^ It has been shown that the decrease in dose from a beam exiting the patient due to a lack of electronic equilibrium also contributes to this effect. The degree to which this decrease is fully modeled by the dose calculation algorithm used varies,^(5)^ which subsequently may cause the severity of fluence boosting to vary between the different algorithms in clinical use.

Previous studies^1^, ^2^ have suggested the use of a “pretend bolus” (PB), applied in treatment planning but not during the actual treatment, as a method to reduce surface dose and high‐dose regions. This method leads the planning system into believing that the optimized PTV is at a depth within the patient rather than at the skin surface, giving adequate buildup of material to account for the skin sparing effect. This method, however, leads to inaccuracies in delivered doses since a bolus present in planning, but not in treatment, has the potential problem that delivered doses at depth higher than those reported in the planning system.

Tomotherapy allows the delivery of helical intensity‐modulated beams of 6 MV energy, and is the most common system used at our center for the treatment of head and neck cancer. Currently at our center, 5 mm of PB is applied when the PTV extends up to the skin surface on treatments using tomotherapy. An alternative method is to reduce the extent of the PTV such that it always remains a certain distance from the surface of the skin (“clipping” the PTV). Such clipping can, in principle, be combined with a postoptimization extension of the beam shape to account for the reduction in the extent of the PTV.^(6)^ However, this functionality is not available with the TomoTherapy planning system, and so clipping inevitably results in a reduced CTV–PTV margin.

The aim of this study is to determine whether alternative strategies of thinner pretend bolus or PTV clipping succeed in controlling the intensity‐modulated fluence within tomotherapy treatments, as measured by the resulting dose heterogeneity and maximum and minimum doses seen in the CTV, as effectively as the 5 mm PB technique.

## II. MATERIALS AND METHODS

### A. Phantom preparation, volume definition, planning and verification

Contours were created in ProSoma (Medcom Ltd, Damstadt, Germany), using the tomotherapy (TomoTherapy, Madison, WI) cylindrical (‘Cheese’) QA phantom of diameter 30 cm and length 18 cm, which is used for testing helical delivery. The phantom was scanned using a GE HiSpeed Nx/i Pro (GE Healthcare, Little Chalfont, UK) with a 3 mm slice width. The image was imported into the ProSoma planning system. A CTV was added 5 mm below the phantom skin surface, consisting of a cylinder of approximately 8 mm diameter and length 48 mm. The PTV was constructed by symmetrically extending the CTV 5 mm in the anterior–posterior (AP), left–right (LR) direction, and 6 mm in the superior–inferior (SI) direction.

**Figure 1:**
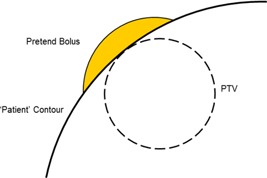
Application of pretend bolus.

Pretend bolus was created by firstly extending the PTV by the desired PB amount, subtracting the phantom skin contour and then changing the density of the resulting structure to match that of the phantom in the TomoTherapy planning system. This effectively changes the shape of the phantom within the planning system, so that optimization is performed as if the radiological path length to the PTV was greater. Figure 1 shows the relative location of the PTV and phantom surface, together with the shape and position of PB.

**Figure 2:**
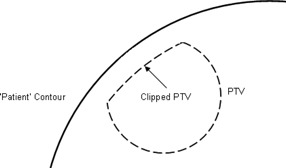
Clipping superficial PTV.

The clip technique was achieved simply enough by reducing the extent of the PTV such that the distance to the skin is, at maximum, the desired amount. This was performed in such a way that the PTV size was reduced asymmetrically back from the skin, and not reduced symmetrically within the phantom (see Fig. 2).

The finite size of the voxels used in the calculation means that the actual radiological depth experienced by voxels optimized as target will vary slightly from place to place.

Initially nine plans were analyzed, four using pretend bolus, four using a clipped PTV, and one with no modification. After initial investigations of the effects of both techniques performed separately, tests were conducted on a further three plans that used combinations of both techniques. The different sizes for PB and Clip extents were 5 mm, 4 mm, 3 mm, and 2 mm. The combinations of PB and Clip used in the final three plans are shown in Table 1.

**Table 1:** Combinations of clipping and pretend bolus.

	*Clip (mm)*	*Pretend bolus thickness (mm)*
Combination 1	2	2
Combination 2	3	2
Combination 3	2	3

### B. TomoTherapy treatment planning

TomoTherapy Hi·Art (Accuray, Sunnyvale, CA) uses dynamic penalized optimization using dose calculations based on collapse cone convolution algorithms. Before optimization, a dose of median 60 Gy to the PTV was prescribed, using a field width of 2.5 cm, pitch of 0.43 on a fine calculation grid, and a modulation factor of 2; final dose calculations were set to be completed after 50 iterations for each case. The fine calculation grid produced a voxel spacing of 2.15 mm.

### C. Plan analysis

**Figure 3:**
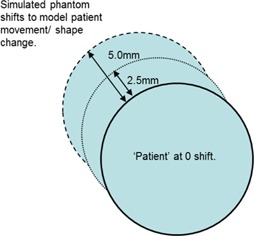
Simulation of patient setup error.

The TomoTherapy DQA (Delivery Quality Assurance) module was used to model the impact of varying phantom position on the delivered dose, as it permits recalculation of a pre‐existent treatment sinogram on an arbitrary phantom shape and position. In order to simulate patient movement, the recalculations were carried out with the phantom situated in three different positions — at the planned position (‘0.0 mm’), and displaced outwards by 2.5 mm and 5.0 mm. The maximum displacement of 5 mm was chosen as this is the magnitude of the CTV–PTV margin performed in our center for head and neck treatments. A shift of 2.5 mm was chosen as the intermediate position between the planned and extreme positions. Only one direction is required for this analysis — that which positions the target as far out towards the edge of the patient as possible (see Fig. 3). Moves in other directions which do not achieve this would not be expected to produce dose increase due to the fluence‐loading effect, nor will they maximize the impact of the reduced CTV–PTV margin produced by clipping, as this chosen direction does. To confirm this expectation, the phantom in the 5 mm PB and 5 mm PTV clip plans was additionally displaced 5 mm inwards, the opposite direction to the remainder of the study.

These simulations were conducted as if the patient (with varying setup error) was actually being treated, so the PB was not included in these recalculations. The resulting dose distributions were studied to determine the minimum dose to the CTV, which was assumed to have moved in tandem with the phantom and, therefore, now at the periphery of the original PTV, and the maximum dose anywhere within the phantom. The baseline measurement for each plan was taken as the results that were obtained for the planned position (0 mm displacement), and the differences from the results at 0 mm displacement to 2.5 mm and 5.0 mm were recorded.

## III. RESULTS

### A. Pretend bolus

**Figure 4:**
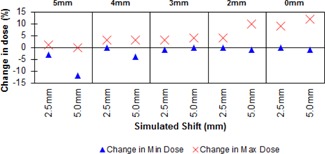
Change in minimum and maximum dose for different thicknesses of PB.

The changes in minimum and maximum doses upon simulated displacement observed in the PB cases are presented in Fig. 4. These are expressed as changes relative to the original planned position for the plan in question. The use of PB generally gives good coverage of the CTV when phantom movement is simulated (average 97.33% SD 0.98 over all shifts and PB thickness); however, notable increases in maximum dose were observed for smaller thicknesses of PB. The maximum dose seen was 119% of prescription, which was for 2 mm PB and a shift of 5 mm, corresponding to an increase of 14% in maximum dose. The 5 mm PB gives the most robust plan, having a modest hotspot of 104% at 5 mm shift whilst still maintaining dose homogeneity in the CTV through all simulated shifts.

### B. PTV clipping

**Figure 5:**
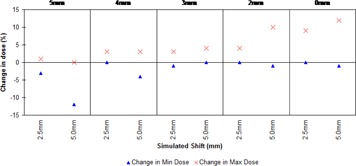
Change in minimum and maximum dose for different amounts of PTV clip.

The results for plans where the PTV was clipped (see Fig. 5) reveal that greater clip distances ameliorate the increase in maximum dose, just as the application of PB does. It was noted, however, that the use of the larger clip distances result in reductions in the minimum dose to the CTV when the phantom has a simulated shift applied. The 5 mm clip gave optimum results in terms of high dose; however, the minimum dose to the CTV was observed to fall from 99% of prescription dose to 87% upon a 5 mm shift. The 4 mm clip gave better coverage, although the maximum dose seen was greater than for the 5 mm clip (107%). The use of 3 mm and 2 mm clips both gave better CTV minimum doses (mean 97.66% and 98.67%, respectively, for all shifts SD 0.57 for both); however, both gave large maximum doses when the patient was shifted (3 mm clip: maximum 110% for 2.5 mm shift; 2 mm clip: maximum 113% for 5 mm shift).

### C. Combinations of both PB and clipping

**Figure 6:**
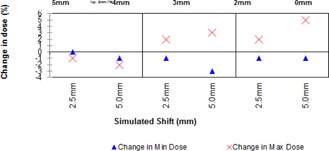
Change in minimum and maximum dose for different amounts of PB and PTV clip combinations.

Following the preliminary findings for different amounts of PB and PTV clip, we decided to investigate whether combinations of both could produce better results. From our initial data, it would appear that having a buildup of approximately 4–5 mm between the PTV and phantom surface (whether the surface be PB or the “real” phantom surface) gave us the best results in terms of minimizing the maximum dose seen when shifts were applied. We therefore decided to use combinations of clip and PB that corresponded to a buildup of approximately 4–5 mm before the PTV, resulting in three plans: 2 mm PB and 2 mm clip, 3 mm PB and 2 mm clip, 2 mm PB and 3 mm clip. The results are shown in Fig. 6.

Overall, the combinations of clipping and PB gave good coverage of the CTV (mean 96% SD 1.4% over all shifts for all plans), and the highest region of maximum dose seen was less than the highest for PB and clipping used separately (110% for 2 mm clip +3mm PB). The combination of 2 mm clip and 2 mm PB gave no increase in the maximum dose regions when the phantom was moved. The 3 mm clip and 2 mm PB combination had poor coverage when the phantom was maximally displaced, and had a maximum dose of 109% compared to 2 mm clip and 3 mm PB, which maintained good coverage when the patient was moved but had a high dose of 110% at the maximum phantom displacement of 5 mm.

A plan with no correction techniques was tested, and is presented in the figures as 0 mm PB and 0 mm clip. This plan showed a maximum high‐dose point of 126% at maximum shift and a minimum of 97% in the PTV.

### D. Inward shifts

An inward shift of 5 mm applied to the 5 mm PB plan increased minimum dose by 3% and increased the maximum dose by 2%, compared with the zero shift calculation. The same shift applied to the 5 mm PTV clip plan decreased the minimum dose by 2% and left the maximum dose unchanged.

## IV. DISCUSSION

Fluence boosting effects occur as inverse treatment planning systems try to compensate for a lack of buildup region, in situations where the PTV extends up to or near the surface of the patient, by boosting beamlet fluence tangential to the superficial region. When the phantom is displaced from its planned position, areas of increased dose near the phantom surface are produced, as exemplified by the plan with no correction strategies suffering regions of high dose up to 126% of prescription near the skin surface upon movement.

In this study, we have looked at different methods of reducing fluence effects in the buildup region on tomotherapy head and neck patients using the TomoTherapy inverse planning system, and have studied the effects of simulated patient movement on the dose distribution.

The methods of using PB and clipping the PTV showed large reductions in fluence boosting when compared to no intervention at all. The coverage of the CTV when no corrections were applied is generally better than for the other methods tested, although only the 5 mm PTV clip method produced a definitively unacceptable minimum dose to the CTV of 87% of the prescription dose when the phantom had a simulated shift of 5 mm applied. The use of combinations of 2 mm clip +2mm PB and 3 mm clip +2mm PB gave slightly less than optimal coverage at maximum shift (94% and 93% regions of low dose, which were considered as anything below 95% of the prescription dose inside the PTV).

When used clinically, the use of PB ‘tricks’ the planning system into carrying out its optimization with the PTV at an adequate depth within the patient, negating the need for fluence boosting at oblique angles. However, as there is, in fact, no real material present when the patient goes to treatment stage, there will be some dosimetric inaccuracy associated with the technique. With this in mind, we tested using variable thicknesses of PB on a cylindrical phantom with the aim of implementing smaller thicknesses in clinical practice. Our results show that reducing the amount of PB used leads to an increased maximum dose, and it was found that the currently used 5 mm PB gave superior results, maintaining good coverage and having a maximum dose of only 104% (at 5 mm shift) of prescription. The other PB thicknesses showed increases in maximum dose of greater magnitudes as the amount of PB was reduced, as a consequence of the amount of buildup seen by the planning system also being reduced.

Patients immobilized in a mask will already have some additional density positioned adjacent to the skin. This is typically too thin to eliminate fluence boosting, but its presence on the patient during treatment will reduce the dosimetric inaccuracy introduced by the use of pretend bolus. Conversely, patients whose weight loss results in a reduction in radiation path length to the target will exacerbate the dosimetric inaccuracy associated with use of the pretend bolus.

The increase in dose at depth due to the removal of planning bolus depends upon the thickness and extent of planning bolus required, which itself varies significantly from patient to patient. In the case of the 5 mm PB plan presented here, that increase is 0.5%. In our practice, plans with considerable quantities of PB are recalculated without PB to determine the likely impact on the delivered dose to the patient.

**Figure 7:**
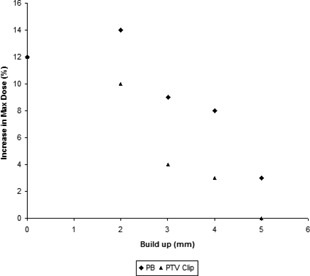
Comparison of different amounts of buildup using PB and PTV clip, and the subsequent maximum dose seen.

**Figure 8:**
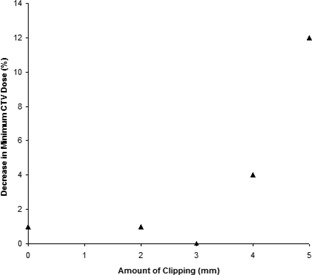
Decrease in minimum dose that covers the CTV with different amounts of clipping.

The degree to which the maximum dose increased upon a shift being applied is correlated with the effective depth of the PTV beneath the phantom surface as experienced by the planning system (Fig. 7). Examining the decrease in minimum dose upon simulated movement of a clipped PTV plan (Fig. 8) reveals that the decrease is, as expected, most evident for greater clip distances, which reduce the CTV–PTV margin considerably.

Following the results obtained for PB and clipping, it was decided to try combinations of the two to get an appropriate level of buildup to see if we could achieve the decrease in hotspots whilst maintaining good CTV coverage. The variation in the increase in maximum dose between the three trials tested is poorly understood, and strategies of mixing PB and clipping appear to have no advantage over purely PB or clipped techniques.

The tendency of IMRT treatment planning systems to deliver greater fluence to superficial parts of the target is, fundamentally, a limitation imposed by the static‐patient approximation used in current systems. Nguyen et al.^(3)^ suggested a “Multiple‐Isocentre CTV‐Based objective function (miCTV)”, performing optimization on a set of potential patient positions, averaging over a sphere with radius equal to the CTV–PTV margin. The use of this method implicitly considers areas of increased dose resulting from fluence boosting effects, without recourse to PB or PTV clipping.

This study primarily focused on changes that can be implemented at the optimization stage of treatment in order to reduce the amount of hotspots in cases where the PTV extends up to the skin surface using the TomoTherapy inverse treatment planning system. The findings here are not obviously extendable to other treatment techniques, such as static‐field IMRT or volumetric‐modulated arc therapy. In particular, static‐beam IMRT, with potentially a small number of beam entry points into the patient, may be expected to experience fluence‐boosting effects of a quite different nature to tomotherapy treatments.

In cases where the CTV extends up to the skin surface, meaning that the PTV extends beyond it, care must be taken to determine the clinician's wishes in and near the buildup region. If full dose is actually required to the surface, then real bolus should be used.

## V. CONCLUSIONS

No alternative technique was seen to be unconditionally superior to a 5 mm PB technique. A clip distance of 3 mm gave acceptable results, but consideration should be given to the reduction in minimum dose or increase in maximum dose seen in clip distances just 1 mm, less or more respectively.
