# “The optimization of superficial planning target volumes (PTVs) with helical tomotherapy”. [JACMP, 15 (6), 2014]

**DOI:** 10.1002/acm2.12117

**Published:** 2017-06-12

**Authors:** M Ashburner, G S Tudor

[https://doi.org/10.1120/jacmp.v15i6.4560]

Typographical error/misrepresentation of results in the original “Chart 4”,^1^ showing the data for the “Pretend Bolus” (PB) which is actually showing the same data as Chart 5.

The correct chart is:

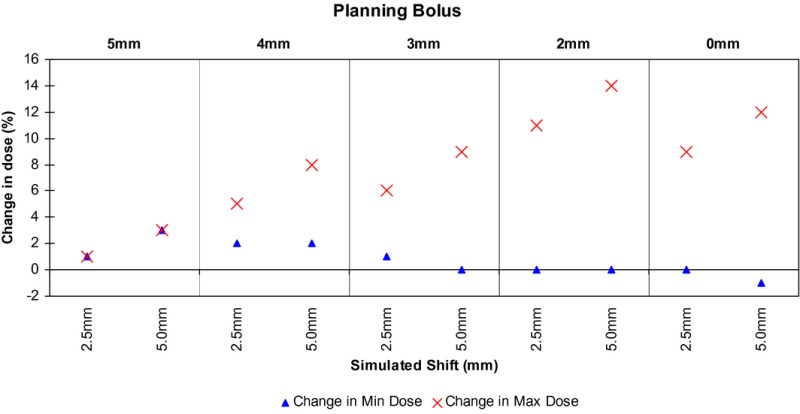

Please note: these are typographical errors whereby initially graph 4 a reproduction of graph 5. The results discussed in the conclusion and other contents and results of the original paper are unaffected.
